# Cardiac arrhythmia detection using cross‐sample entropy measure based on short and long RR interval series

**DOI:** 10.1002/joa3.12839

**Published:** 2023-03-22

**Authors:** Kanchan Sharma, Ramesh Kumar Sunkaria

**Affiliations:** ^1^ Department of Electronics and Communication Engineering Dr B R Ambedkar National Institute of Technology Jalandhar Punjab India

**Keywords:** cardiac arrhythmia detection, cross sample entropy, irregularity, R‐R interval series, sample entropy

## Abstract

**Background:**

Accurate arrhythmia (atrial fibrillation (AF) and congestive heart failure (CHF)) detection is still a challenge in the biomedical signal‐processing field. Different linear and nonlinear measures of the electrocardiogram (ECG) signal analysis are used to fix this problem.

**Methods:**

Sample entropy (SampEn) is used as a nonlinear measure based on single series to detect healthy and arrhythmia subjects. To follow this measure, the proposed work presents a nonlinear technique, namely, the cross‐sample entropy (CrossSampEn) based on two series to quantify healthy and arrhythmia subjects.

**Results:**

The research work consists of 10 records of normal sinus rhythm, 20 records of Fantasia (old group), 10 records of AF, and 10 records of CHF. The method of CrossSampEn has been proposed to obtain the irregularity between two same and different R–R (R peak to peak) interval series of different data lengths. Unlike the SampEn technique, the CrossSampEn technique never awards a ‘not defined’ value for very short data lengths and was found to be more consistent than SampEn. One‐way ANOVA test has validated the proposed algorithm by providing a large F value and *p* < .0001. The proposed algorithm is also verified by simulated data.

**Conclusions:**

It is concluded that different RR interval series of approximate 1500 data points and same RR interval series of approximate 1000 data points are required for health‐status detection with embedded dimensions, *M* = 2 and threshold, *r* = .2. Also, CrossSampEn has been found more consistent than Sample entropy algorithm.

## Graphical abstract

The first step of the research work is to extract RR intervals are extracted from ECG signals and utilize pre‐processing techniques to banish outliers from data. The outlier‐free data is utilized to evaluate entropy. In the research work, linear interpolation is utilized to remove outliers. Entropy values indicate the detection of arrhythmia subjects.

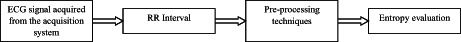

## 1 INTRODUCTION

Different biological systems have complex structures and behaviors.^1^ The cardiovascular system is one of them and it consists of the heart and the circulatory system. The heart's faulty signaling makes it beat too fast, too slow or irregularly and this is known as arrhythmia. Accurate arrhythmia (congestive heart failure (CHF)^2^ and atrial fibrillation (AF)) detection is essential to make patients' life better as it prevents unexpected deaths.^3^ There are different methods to detect arrhythmia and types of arrhythmia. But accurate detection of arrhythmia is still a goal to achieve in the biomedical signal‐processing field.^3^ Electrocardiogram (ECG) analysis uses linear and the nonlinear methods to find the hidden information and these can be used to detect arrhythmia.^4^ Nonlinear techniques of biomedical signal analysis are preferred over linear techniques to extract the hidden information, as they are more accurate than linear techniques.^4^ Nonlinear techniques are approximate entropy (ApproxEn), sample entropy (SampEn), and cross‐sample entropy (CrossSampEn) etc.^4^

RR interval is one of the critical features of the ECG signal which is used to quantify heart rate variability (HRV).^5^ Moreover, RR intervals are sensitive to detect physiological and pathological subjects.^5^ For the detection of physiological and pathological subjects, a technique known as entropy is used. Entropy is a very informative tool and can find the hidden information of any signal. This hidden information tells about whether a person has heart disease or not.^6^ It has been found that healthy subjects have less irregularity and more complexity and pathological persons have less complexity and more irregularity.^7^, ^8^, ^9^ One of the most popular entropy algorithms is ApproxEn, but lack of relative consistency and dependence on data length are two important limitations of ApproxEn.^10^, ^11^ To overcome its negative points, a SampEn algorithm is developed to obtain the value of irregularity or complexity of a RR interval series^12^ and it requires spikes‐free ECG data before computation.^13^ SampEn has also found one of its applications to classify sleep stages with restricted channels.^14^ There is another entropy method, known as CrossSampEn, a non‐linear measure, used to obtain the irregularity and complexity of two RR interval series instead of one RR interval as in case of the SampEn.

To understand the concept of CrossSampEn, Liu et al. showed that CrossSampEn is better as compared to the correlation between interval series.^15^ Wenbin Shi et al. employed CrossSampEn to measure the dissimilarity for stock markets.^16^ Multiscale Cross Trend SampEn (MCTSE) is used to find asynchrony for two series but at multiple scales.^17^ Jamin et al. reviewed a paper on cross‐entropy and multiscale cross‐entropy methods to find the asynchronism between two‐time series.^18^ Cross sample entropies use deep learning model to reveal complexity‐related data series and functional connectivity between areas of the brain.^19^ Bonal et al. completed the aspects of ApproxEn, CrossApproxEn, SampEn, and CrossSampEn algorithm based on theory and applications of various fields.^10^ Fabris et al. assessed the voice disorder based on SampEn and CrossSampEn algorithms for electroglottogram and microphone signals.^20^ CrossSampEn is utilized for two different and same RR interval series to obtain the value of cross dissimilarity and it is based on SampEn.^6^ There are some methods of entropy analysis and their use in the examination of biomedical signal.^22^ The SampEn algorithm was introduced by Richman et al.^3^, ^21^, ^23^ to identify the irregularity of a time series and it is used to detect arrhythmia. To decrease unexpected death rate owing to arrhythmia, the proposed algorithm, CrossSampEn also plays a major role in the biomedical signal‐processing field. The proposed algorithm, CrossSampEn, has its application for arrhythmia detection with small data length as SampEn is invalid to work with small data length.

In the research work, CrossSampEn algorithm is proposed and it is remarked that it never awards a ‘not defined’ value for any data length (*N*) as it occurs in the case of SampEn.^24^, ^25^ A new observation is observed by using CrossSampEn that it is an effective nonlinear measure for two similar RR interval series with data length scales from 10 and above and is effective for different series of data length scaling from 1500 data points with embedded dimension, *M* = 2 and threshold, *r* = .2. The other purpose of this research is to select the arrhythmia patient from (1) the arrhythmia patients' group only, (2) healthy and arrhythmia patients' group, and (3) compare two same subjects. The proposed work is evaluated with simulated data and one‐way ANOVA test. Both find that results are constructive.

## 2 METHODOLOGY

**FIGURE 1:**
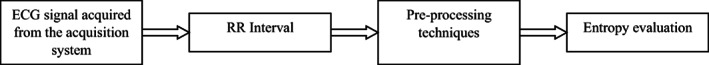
Block diagram representation.

The proposed research methodology is shown in the block diagram given in (Figure 1):

The first step of this research is to extract RR intervals from ECG signals and utilize pre‐processing techniques to remove outliers from data.^5^ The outlier‐free data are utilized to evaluate entropy^5^ and are presented in Figure 1. In this research, linear interpolation is utilized to remove outliers. SampEn and CrossSampEn are two entropy measurement techniques that are discussed in this research.

### 2.1 Sample entropy algorithm

The steps of SampEn algorithm are given below^3^, ^10^, ^26^

1. Let RR interval series be

(1) $$u\left(i\right)=m\left(i\right)+m\left(i+1\right)+m\left(i+2\right)..\ldots \ldots m\left(i+M-1\right)$$

where i=1,2⋯N−M+1 and form $U^{M}\left(i\right)$ vectors, *M* is the embedded dimension, and *N* is the data length. This RR interval series should be free from outliers.^27^ The important concept is that this RR interval series must be standardized to 1.

2 The distance can be calculated as

(2) $$d\;\left(u,v\right)=\max\;\left(m\left(i+k\right)-n\left(j+k\right)\right)$$

where k=0≤k≤M−1. Measure each element of the series by finding the difference between the scalar components of these vectors.

3 $U_{r}^{M}\left(i\right)$ considered as the probability for templates $U^{M}\left(i\right)$ and $U^{M}\left(j\right)$ to come within the threshold value, *r*

(3) $$U_{r}^{M}\left(i\right)=N^{M}\left(i\right)\left(\frac{1}{N-M+1}\right)$$

4 Calculate $U\left(i\right)$ by using

(4) $$U\left(i\right)=\left(\frac{1}{N-M}\right)\sum_{i=1}^{N-M}U_{r}^{M}\left(i\right)$$

The above steps are for embedded dimension *M*.

5 For embedded dimension *M* + 1, calculate

(5) $$V\left(i\right)=\left(\frac{1}{N-M}\right)\sum_{i=1}^{N-M}V_{r}^{M}\left(i\right)$$

where $V_{r}^{M}\left(i\right)=\left(\frac{1}{N-M}\right)\sum_{i=1}^{N-M}W_{r}^{M}\left(i\right)$, $W_{r}^{M}\left(i\right)$ measuring the distance between scalar elements of the vectors and compare them with the threshold value, *r* by rejecting the self‐matching criteria. The distance formula is $d\;\left(u,v\right)\leq r$ .

6 The Sample Entropy (SampEn) is^28^, ^29^:

(6) $$\text{SampEn}=-\ln\left(\frac{V\left(i\right)}{U\left(i\right)}\right)$$

### 2.2 Cross sample entropy

CrossSampEn algorithm is based on the SampEn algorithm^6^, ^15^ and originally introduced by Pincus.^30^ The limitations of the SampEn are eliminated in the CrossSampEn algorithm. The steps of the CrossSampEn algorithm are given below^6^, ^15^:

1. Let the two‐time series are

(7) $$u\left(i\right)=m\left(i\right)+m\left(i+1\right)+m\left(i+2\right)\cdots m\left(i+M-1\right)$$

$$v\left(i\right)=n\left(j\right)+n\left(j+1\right)+n\left(j+2\right)\cdots n\left(j+M-1\right)\left(8\right)$$

where i=1,2…N−M+1 and j=1,2…N−M+1. *M* is the embedded dimension and *N* is the data length.

2 The distance can be calculated as

(8) $$d\;\left(u,v\right)=\max\;\left(m\left(i+k\right)-n\left(j+k\right)\right)$$

where k=0≤k≤M−1.

3 Calculate $U_{i}\left(M\right)$ by using

(9) $$U_{i}\left(M\right)=\left(\frac{1}{N-M+1}\right)\sum_{i=1}^{N-M}\left[N_{i}\left(M\right)\right]$$

$N_{i}$(*M*) indicates the number of matches found by comparing the distance between elements of two series and compare them with the threshold value, *r*. The matching condition is $d\left(u,v\right)\leq r$.

4 Calculate $U\left(M\right)$ by using

(10) $$U\left(M\right)=\left(\frac{1}{N-M}\right)\sum_{i=1}^{N-M}\left[U_{i}\left(M\right)\right]$$

The above steps are for embedded dimension *M*.

For embedded dimension M + 1, calculate

(11) $$V\left(M\right)=\left(\frac{1}{N-M}\right)\sum_{i=1}^{N-M}\left[W_{i}\left(M\right)\right]$$

$W_{i}\left(M\right)$ indicates the number of matches found by comparing the distance between the elements and compare them with the threshold value, *r* for embedded dimension *M* + 1. The matching condition is $d\;\left(u,v\right)\leq r$.

5 The CrossSampEn is

(12) $$\text{CrossSampEn}=-\ln\left(\frac{V\left(M\right)}{U\left(M\right)}\right)$$

### 2.3 Parameters and selection

It is very important to choose parameters of SampEn^24^ and CrossSampEn with caution.^31^ It was observed that the reliability of the short dataset is more in the case of the SampEn^24^, ^32^ and awards a ‘not defined’ for very short data length. In the CrossSampEn algorithm, the threshold value, *r*, is predetermined to be .2, as it is good to classify healthy and arrhythmia subjects' groups, classification of arrhythmia subjects' group only, and compare same subjects. The embedded dimension, *M*, should be 2 or 3. In the algorithm of this present work, *M* = 2 is considered. The number of data points is considered up to 2000.

### 2.4 Data

The data are taken of RR intervals of different databases from Physionet.org ^33^ and few data records are presented in this paper. The software used in the present work is MATLAB 2017b. The data records considered are of normal sinus rhythm (NSR), AF from MGH/MF database, also taken CHF and Fantasia (old group) database. There are 10 records of NSR, 20 records of Fantasia (old group), 10 records of AF, and 10 records of CHF and are presented in the research. In both databases NSR and AF sampling intervals were 360 Hertz (Hz). The sampling interval of CHF is 128 Hz and Fantasia is digitized at 250 Hz. All these records are of subjects having ages 60 years and above. The traditional SampEn algorithm and CrossSampEn algorithm are applied in these databases to obtain the value of asynchrony and cross asynchrony, respectively. For collecting data, a 1‐h recording was taken for each database and from the recording, up to 2000 sample points (sample size) were considered. It is important to mention that CrossSampEn of the same subjects' outcome is of one data record only, whereas for different subjects, each record of one database is compared with multiple records of another database. The current study is exempt from ethical approval as the work was performed on standard databases from Physionet.org not on humans and animals.

### 2.5 Simulated data

To confirm this research algorithm, synthetic signals are utilized which are generated in MATLAB 2017b. The synthetic signals consist of periodic sine and cosine waves, *N* is considered 10 to 10 000 and *M* = 2. Also CrossSampEn algorithm was compared with SampEn algorithm.

### 2.6 Statistical data

The data are presented in the form of mean ± standard error. One‐way ANOVA test was used to compare SampEn and CrossSampEn algorithms and the results were significant if large F value and p≤.0001 met.

## 3 RESULTS

### 3.1 Arrhythmia and healthy subjects

**FIGURE 2:**
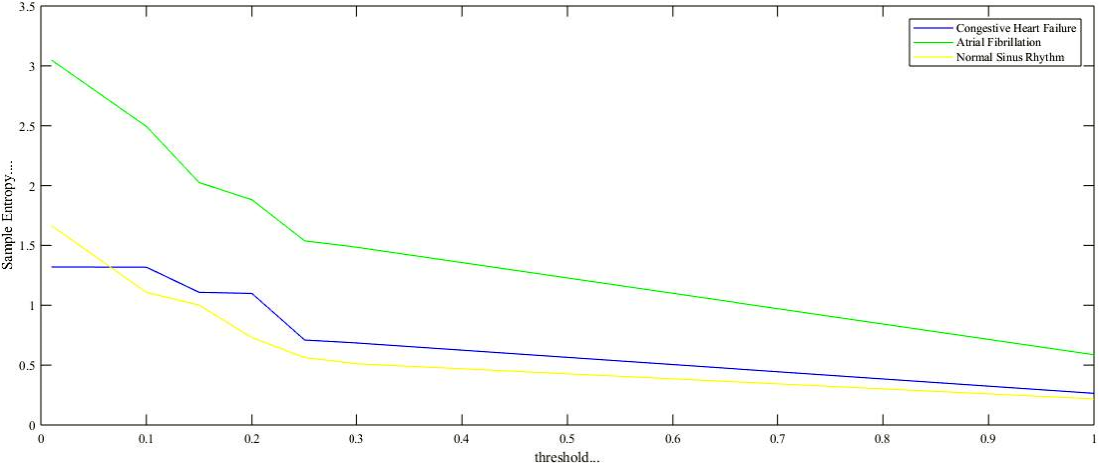
SampEn of different subjects as a function of threshold, *r*. By increasing the value of threshold, SampEn reduces and reaches the nearest zero. The threshold value, *r* is chosen as 0.2 because this threshold provides the best discrimination among NSR, CHF and AF.

The traditional SampEn algorithm is used to obtain irregularity^5^, ^11^ of a time series. In the present research, it is observed and verified that healthy subjects (NSR) have less irregularity than arrhythmia subjects (CHF and AF).^34^ Hence, the SampEn of the arrhythmia subjects are more compared to the SampEn of healthy subjects as shown in Figure 2.

Figure 2 reveals the value of the SampEn for *r* = .01, .1, .15, .2, .25, and shows the discrimination among NSR, CHF, and AF on the basis of the SampEn. As the value of threshold *r*´ increases, the SampEn is getting lower (nearest to zero) as shown in Figure 2.

To obtain the results of CrossSampEn, each record of a healthy subject (NSR and Fantasia) is crosschecked with each record of considered arrhythmia subject (CHF and AF) and it shows how irregular an arrhythmia patient is as compared to healthy subjects. For the evaluation of different series, 20 records of Fantasia (old group) are compared with 10 records of CHF, and 10 records of AF and 10 records of NSR are compared with 10 records of AF, and 10 records of CHF are shown in Table 2. It is further determined that the CrossSampEn of NSR/Fantasia—AF is more as compared to NSR/Fantasia—CHF as NSR/Fantasia—AF has more irregularity than the NSR/Fantasia—AF.

### 3.2 Arrhythmia subjects' group

The other information that was observed from the proposed work is that the subjects with AF has more irregularity than the subjects with CHF. Hence, the SampEn of AF is more compared to the SampEn of CHF. SampEn is less in the case of NSR as shown in Table 1 and Table 2. It has been noticed that CrossSampEn also shows less irregularity for CHF than AF shown in Table 2. For the evaluation of arrhythmia group, 10 records of AF are compared with 10 records of CHF and its irregularity is shown in Table 2.

**TABLE 1:** Traditional SampEn of “Normal Sinus Rhythm, NSR”, “Atrial Fibrillation, AF” and “Congestive Heart Failure, CHF”.

S. No	NSR (mean ± SE)	AF (mean ± SE)	CHF (mean ± SE)
1.	0.7303 ± 0.0256	1.8850 ± 0.0446	1.0990 ± 0.0455

**TABLE 2:** Comparison chart of SampEn and CrossSampEn analysis (same and different subjects) in terms of mean with standard error (SE).

S. No	Database 1	Database 2	CrossSampEn (mean ± SE)
1.	NSR	NSR	0.7891 ± 0.1461
2.	NSR	AF	1.9544 ± 0.0766
3.	NSR	CHF	0.9577 ± 0.1037
4.	Fantasia	AF	1.9002 ± 0.0278
5.	Fantasia	CHF	1.3238 ± 0.0940
6.	AF	AF	1.7886 ± 0.0746
7.	AF	CHF	1.8602 ± 0.0855
8.	CHF	CHF	1.1512 ± 0.1238

	Database	SampEn (mean ± SE)
1.	NSR	0.7303 ± 0.0256
2.	CHF	1.0990 ± 0.0455
3.	AF	1.8850 ± 0.0446

### 3.3 Same subjects' group

It has been observed that CrossSampEn of NSR and AF group, NSR and CHF group are more than the CrossSampEn of the group of same subjects. The reason for this is that dissimilarity is more for records of different databases compared to the same subjects of same databases. It has been verified that two RR interval series are synchronous to each other, less is CrossSampEn, and if two RR interval series are asynchronous to each other, CrossSampEn is more.^6^, ^15^

It is important to mention that CrossSampEn of two same subjects considers two same records only, whereas for different subjects, one record of one database is compared with multiple records of another database shown in Table 2. The group of the same subjects includes 10 records of AF, 10 records of CHF, and 10 records of NSR.

### 3.4 Simulated series and real data

**FIGURE 3:**
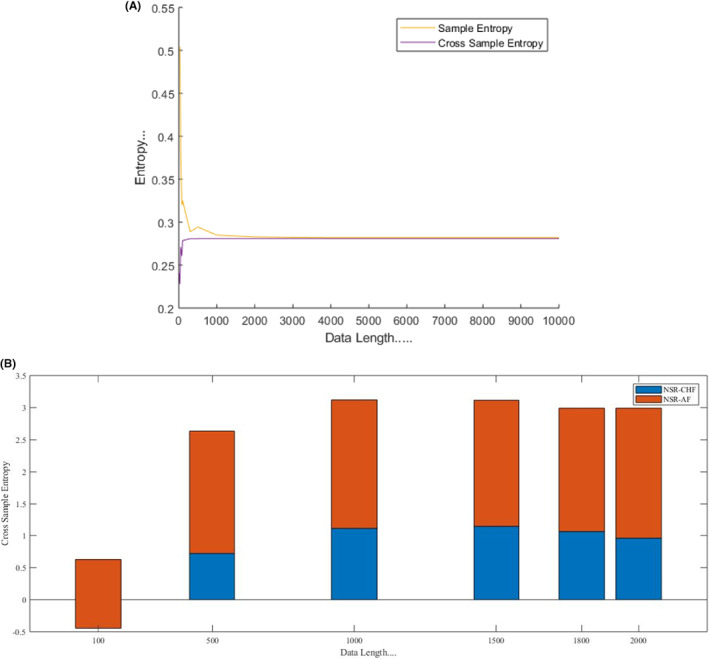
(A) CrossSampEn (two same series simulated data) and SampEn analysis with respect to data length. It has been found that CrossSampEn is more consistent than SampEn. The CrossSampEn is stabilized for the data length 10 and above, more stabilized for the data length 1000 and above, whereas SampEn is stabilized for the data length 500 to 4000 and more stabilized for the data length 4000 and above. (B) CrossSampEn analysis of NSR‐CHF group and NSR‐AF group (two real data different series) for different data lengths. Blue bars represent NSR‐CHF group and orange bars represent NSR‐AF group. The figure shows that the best discrimination between NSR‐CHF group and NSR‐AF group has been found for the data length 1500 and above with good consistency.

**FIGURE 4:**
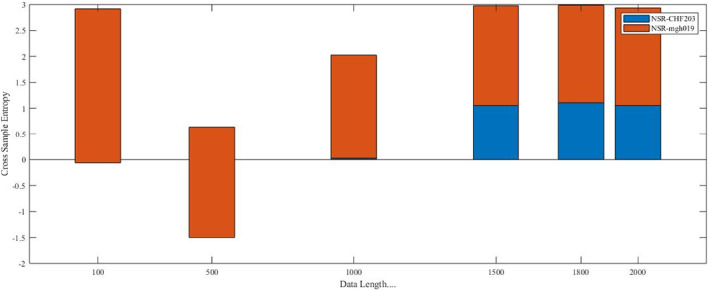
CrossSampEn analysis of NSR records with AF record, mgh019 and CHF record, chf203 record. It has been shown here that good consistency has been achieved by CrossSampEn for data length 1500 and above. CrossSampEn provided no ‘not defined’ value for any data length,

Numerous tests are conducted on the simulated data as well as on real data to explore the exactness of CrossSampEn algorithm for different data lengths. The data length is considered from 10 to 10,000 for same simulated data with threshold, *r* = .2 and *M* = 2 as shown in Figure 3 and data length 200 to 2000 is considered for two different series real data with threshold, *r* = .2 and *M* = 2 as shown in Figure 4.

It has been observed that SampEn algorithm works well for *N* ≥ 500 for same simulated data. But for dataset with *N* < 500, SampEn does not award stabilized result. It is noticed that CrossSampEn does not follow this and performs well for two same RR interval series with data length scales from 10 and above shown in Figure 3; results are not appropriate for different series with data length, *N* < 1500; never awards a ‘not defined’ value. The quantification of real data with two different series (healthy and arrhythmia subjects' group) with data length 100 to 2000 is shown in Figure 4.

Figure 3A shows the connection between data length and both SampEn and CrossSampEn (two same series) values based on the simulated data. Here, SampEn is utilized to investigate the irregularity of a signal, and the CrossSampEn is utilized to compare two same signals. It has been explored that for embedded dimension, M=2 and threshold, $r=.2*\mathrm{std}\left(\text{data}\right)$, SampEn algorithm performs well for data length 500 to 4000; the graph is stabilized for this data length; more stabilized for data length 4000 and above, whereas CrossSampEn algorithm performs well for two same RR interval series with *N* < 1000 scales from 10; the graph is stabilized for this data length, and more stabilized for data length 1000 and above as shown in Figure 3A. It is also determined that CrossSampEn algorithm is less delicate to the data length. According to the research work, SampEn awards a ‘not defined’ for some small data length (when the identification of regularity is nil and the conditional probability is nil) and CrossSampEn algorithm conquers this shortcoming of SampEn; never results a ‘not defined’ for any data length shown in Figure 3A,B. Therefore, it is important to concentrate on the mingling of *M*, *r*, and *N* as both algorithms are delicate to the mingling of *M* and *r* but less delicate to *N*.

Figure 3B shows the outcomes of real data different series (healthy and arrhythmia patients) with data length 100 to 2000 by using CrossSampEn algorithm and it is newly observed that approximate 1500 data points are essential to differentiate healthy and arrhythmia patients with embedded dimension, *M* = 2 and threshold, *r* = .2. To show the consistency of different series and to investigate the ‘not defined’ value of the same with CrossSampEn algorithm, record chf203 of CHF and record mgh019 of AF from physionet.org with the data length ranging from 100 to 2000 is compared with NSR and got new outcome that for two different series of any data length, CrossSampEn algorithm has no ‘not defined’ value as existed in the SampEn algorithm for some small data length (when the identification of regularity is nil and the conditional probability is nil) and to quantify healthy and arrhythmia patients, the outcomes are consistent for the data length ranging from 1500 and above as shown in Figure 4.

**FIGURE 5:**
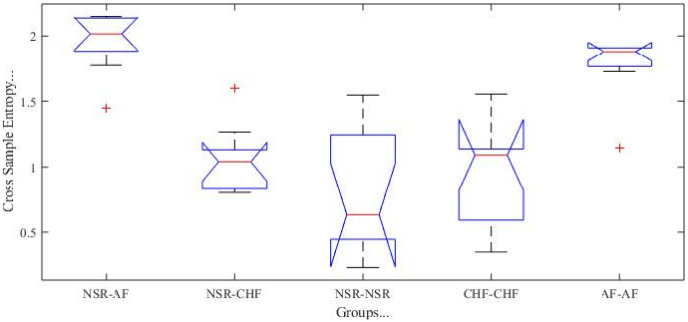
Quantification among different groups (NSR, CHF, and AF) with CrossSampEn. One‐way ANOVA test has made this comparison by providing very significant *p* value (<.0001) and large F value. 10 records of NSR, 10 records of AF and 10 records of CHF are compared with each other to get irregularity of one RR interval series to other.

To validate the success of this research method, the one‐way ANOVA test is conducted in subjects of considered 3 groups. There are 10 subjects of AF, 10 subjects of CHF, and 10 subjects of NSR. All are included in the old age group (>60 years). The outcome about differentiation among groups is shown in Figure 5 and got a very significant *p*‐value (<.0001). It is important to mention that CrossSampEn of the same subjects' outcome is of two same data record only whereas, for different subjects, each record of one database is compared with multiple records of another database.

**FIGURE 6:**
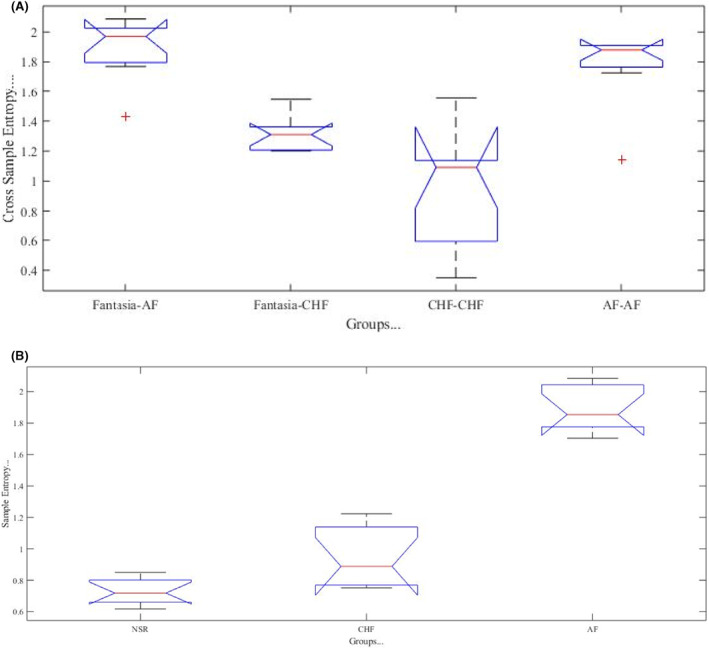
(A) Quantification among different groups (Fantasia, CHF, and AF) with CrossSampEn. One‐way ANOVA test has made this comparison by providing very significant *p* value (<.0001) and large F value. 10 records of Fantasia, 10 records of AF and 10 records of CHF are compared with each other to get irregularity of one RR interval series to other. (B) Quantification among different groups (NSR, CHF, AF) with SampEn. One‐way ANOVA test provides a very significant value of *p* (<.0001) and large F value for the comparison of NSR, CHF, and AF groups. It has been shown by comparing three groups that all are distinguished from each other.

In the same way, one way ANOVA test is conducted for CrossSampEn with 20 records of Fantasia, 10 records of AF, 10 records of CHF, and the outcome gives a validated result with a very significant *p*‐value (<.0001) as shown in Figure 6A. These records are of the older age group. It is important to mention that 20 records of Fantasia are compared with 10 records of CHF and with 10 records of AF.

Furthermore, to validate the success of SampEn algorithm, the one‐way ANOVA test is conducted. It has been found that this algorithm is able to distinguish healthy and arrhythmia subjects. This test validates the result with a very significant *p*‐value (<.0001). These records are of the older age group. It is important to mention that the SampEn algorithm works to differentiate three groups (NSR, AF and CHF) with 10 records each shown in 6B.

## 4 DISCUSSION

Quantitative estimation of HRV based on nonlinear techniques is good to find hidden information, but nonlinear measurement demands a large data set to find entropy.^26^ Entropy is defined as a measure of the irregularity of a system. In this proposed work, the CrossSampEn is used as a non‐linear measure to detect cardiac arrhythmia and compare this algorithm with the SampEn. A nonlinear measure named the SampEn awards a ‘not defined’ when the regularity identification and conditional probability are zero and this is practicable for those cases having very short data length as the numbers of the data points are very less to find an appropriate result. A very new observation is added in this paper is to check the irregularity based on very short data length by using a nonlinear measure named the CrossSampEn. It is concluded that CrossSampEn algorithm never awards a ‘not defined’ for very short data length as in the case of SampEn algorithm; performs well for two same RR interval series having data length scales from 10 and above, but it is failed to perform well for two different RR interval series having data length scales from 200 and above without resulting a ‘not defined’; to differentiate different series (healthy and arrhythmia patients), data length ranges from 1500 data points with embedded dimension, *M* = 2 and threshold, *r* = .2 shown in Figures 3 and 4 respectively. The proposed work is used to differentiate between healthy subjects and arrhythmia patients. It has been concluded that CrossSampEn is more consistent than SampEn.

SampEn is used to find the irregularity of a series whereas CrossSampEn is used to find the irregularity between two similar and different series. It is concluded that the healthy subjects have less irregularity than the arrhythmia subjects. Therefore, the SampEn of NSR is more than that of arrhythmia subjects. On the basis of the irregularity, it is observed that the CrossSampEn of NSR–CHF subjects have less value than NSR‐AF subjects as the irregularity of NSR‐CHF subjects is less than that of NSR–AF subjects. It has been observed that CrossSampEn between two different series are more than two same series.

The irregularity between two series is detected by the threshold parameter, *r* and the CrossSampEn algorithm sets this threshold value, *r* = .2 with embedded dimension, *M* = 2 to differentiate between two same and different series. It is concluded that the CrossSampEn algorithm performs well with this threshold, *r* = .2 with embedded dimension, *M* = 2 to differentiate between two same and different series. The proposed algorithm is compared with the SampEn algorithm to detect cardiac arrhythmia.

Moreover, there is a relation between (HRV^12^ and Autonomic Nervous System (ANS)).^35^, ^36^ Autonomic Nervous System consists of sympathetic and parasympathetic nervous system and HRV is controlled by ANS.^36^, ^37^ Heart rate variability of two subjects is analyzed by CrossSampEn, but the relationship between ANS and CrossSampEn has not been realized yet.

## 5 CONCLUSIONS

The proposed algorithm, named the CrossSampEn, is brilliant to pick out arrhythmia subjects from healthy and arrhythmia subjects' group and arrhythmia subjects' group only. This distinction is based on irregularity and uses two same and different RR interval series at a single time and depends on the value of the threshold, *r* and it is preferable to select 0.2 as a permanent threshold for classification rather than ranging threshold values for different arrhythmia and healthy subjects. It molds the proposed algorithm into a simple algorithm for classification purpose. It was observed that the CrossSampEn is less if one RR interval series is in synchronization with other and more CrossSampEn value if two RR interval series are not in synchronization. Also, CrossSampEn is more consistent than SampEn algorithm.

A very new observation of the CrossSampEn algorithm is that it performs well as a nonlinear measure for two same series with data length scaling 10 and above; minimum 1500 data points with embedded dimension, *M* = 2 and *r* = .2 are required to quantify healthy and arrhythmia patients (different series). This research is compared with the SampEn algorithm as the SampEn awards a ‘not defined’ entropy when the identification of regularity is nil and conditional probability is nil. This exists only in the case of a very short dataset. The CrossSampEn improved the SampEn technique to eliminate this problem and never results in a ‘not defined’ when the identification of regularity and the conditional probability are nil. To examine this algorithm, two one‐way ANOVA tests are conducted and a large F value and p<.001 designate the validity of this algorithm. The proposed algorithm is also verified by using simulated data. The inequality among the same and different series of the same groups and different groups are openly observed.

## FUNDING INFORMATION

The authors declare that no funds, grants, or other support were received during the preparation of this manuscript.

## CONFLICT OF INTEREST STATEMENT

The authors have no relevant financial or non‐financial interests to disclose.

## ETHICS STATEMENT

Not Applicable.

## CONSENT TO PUBLISH

Not Applicable.

## CONSENT TO PARTICIPATE

Not Applicable.

## CLINICAL TRIAL REGISTRATION

Not Applicable.
